# Like a rolling stone: Colonization and migration dynamics of the gray reef shark (*Carcharhinus amblyrhynchos*)

**DOI:** 10.1002/ece3.9746

**Published:** 2023-01-10

**Authors:** Pierre Lesturgie, Camrin D. Braun, Eric Clua, Johann Mourier, Simon R. Thorrold, Thomas Vignaud, Serge Planes, Stefano Mona

**Affiliations:** ^1^ Institut de Systématique, Evolution, Biodiversité (ISYEB), Muséum National d'Histoire Naturelle, EPHE‐PSL Université PSL, CNRS, SU, UA Paris France; ^2^ Biology Department Woods Hole Oceanographic Institution Woods Hole Massachusetts USA; ^3^ Laboratoire d'Excellence CORAIL Papetoai French Polynesia; ^4^ EPHE, PSL Research University Paris France; ^5^ Université de Corse Pasquale Paoli, UMS 3514 Plateforme Marine Stella Mare Biguglia France

**Keywords:** *Carcharhinus amblyrhynchos*, *Carcharhinus melanopterus*, demographic history, meta‐population, Radseq, range expansion

## Abstract

Designing appropriate management plans requires knowledge of both the dispersal ability and what has shaped the current distribution of the species under consideration. Here, we investigated the evolutionary history of the endangered gray reef shark (*Carcharhinus amblyrhynchos*) across its range by sequencing thousands of RADseq loci in 173 individuals in the Indo‐Pacific (IP). We first bring evidence of the occurrence of a range expansion (RE) originating close to the Indo‐Australian Archipelago (IAA) where two stepping‐stone waves (east and westward) colonized almost the entire IP. Coalescent modeling additionally highlighted a homogenous connectivity (*Nm* ~ 10 per generation) throughout the range, and isolation by distance model suggested the absence of barriers to dispersal despite the affinity of *C. amblyrhynchos* to coral reefs. This coincides with long‐distance swims previously recorded, suggesting that the strong genetic structure at the IP scale (*F*_ST_ ~ 0.56 between its ends) is the consequence of its broad current distribution and organization in a large number of demes. Our results strongly suggest that management plans for the gray reef shark should be designed on a range‐wide rather than a local scale due to its continuous genetic structure. We further contrasted these results with those obtained previously for the sympatric but strictly lagoon‐associated *Carcharhinus melanopterus*, known for its restricted dispersal ability. *Carcharhinus melanopterus* exhibits a similar RE dynamic but is characterized by a stronger genetic structure and a nonhomogeneous connectivity largely dependent on local coral reefs availability. This sheds new light on shark evolution, emphasizing the roles of IAA as source of biodiversity and of life‐history traits in shaping the extent of genetic structure and diversity.

## Graphical abstract

*Carcharhinus amblyrhynchos* is an endangered Indo‐Pacific reef shark, which shows reef fidelity but long‐distance movements, raising questions about connectivity patterns and the extent of conservation units. Using genomic data, we show that it has undergone a range expansion from the Indo‐Australian Archipelago (IAA) and is organized as a meta‐population characterized by homogeneous connectivity throughout its range. We highlight the low dependence of *C. amblyrhynchos* on reef availability and its ability to cross open sea expanses, which do not represent barriers to gene flow, contrasting with the sympatric *Carcharhinus melanopterus*, whose dispersal is strictly dependent on local reef distribution. Conversely, both species share similar range expansion dynamics, suggesting a major importance of the IAA as a source of biodiversity for reef sharks.

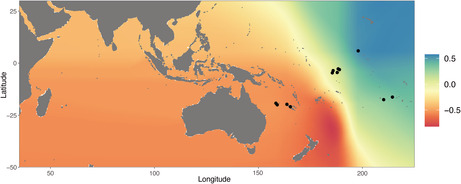

## 1 INTRODUCTION

More than 37% of shark species are currently threatened with extinction (Dulvy et al., 2021) and less than 30% are on stable or increasing population trend according to the International Union for Conservation of Nature (IUCN) Red List of threatened species. As meso or apex predators, they hold important roles in their ecosystems (Bornatowski et al., 2014) and their decline has already shown negative cascading effects on food web structure (Friedlander & DeMartini, 2002; Myers et al., 2007). Although local‐scale conservation programs have been established, their efficiency has been questioned for some species of sharks (Robbins et al., 2006; Speed et al., 2016). For instance, local‐scale management might not always be consistent with the home range size and the dispersal ability of sharks (see Dwyer et al., 2020). Genetics and ecological evidence have identified both species with very restricted home ranges (Mourier et al., 2013; Whitney et al., 2012) and species capable of crossing large expanses of the ocean (Bailleul et al., 2018; Corrigan et al., 2018; Pirog et al., 2019). Designing appropriate management actions is therefore a difficult task requiring the knowledge of both the dispersal ability of the species under investigation and the existence of barriers to gene flow, which are often hard to identify in the marine realm.

Population genomics is becoming increasingly important in this context, particularly because of the large amount of data provided by the emergence of next‐generation sequencing approaches (NGS). It is now possible to assess the genetic diversity of model or nonmodel species at an unprecedented level of accuracy (Benazzo et al., 2017; Steiner et al., 2013). However, genetic diversity alone does not provide clues on the evolutionary trajectory of a species, and a careful modeling is required to fully understand its demographic history and the conservation challenges to be faced. Unfortunately, for computational reasons, many commonly used software implement, under different algorithms, *unstructured* models, i.e., models that consider the population under investigation as isolated or panmictic (Heled & Drummond, 2008; Heller et al., 2013; Li & Durbin, 2011; Liu & Fu, 2015). Except for highly vagile species, which are panmictic at a large scale (Corrigan et al., 2018; Lesturgie et al., 2022; Pirog et al., 2019), broadly distributed sharks species are more likely organized in meta‐population(s) throughout their range (Maisano Delser et al., 2016, 2019; Momigliano et al., 2017; Pazmiño et al., 2018). The application of *unstructured* models to species organized in meta‐populations yields spurious signatures of effective populations size (*N*_*e*_) changes through time (Chikhi et al., 2010; Maisano Delser et al., 2019; Mazet et al., 2016, 2015), with potentially dangerous consequences in terms of conservation policies. However, recent studies have highlighted the usefulness of such models to characterize the gene genealogy of the sampled lineages, which in turn reveals important features of the meta‐population (Arredondo et al., 2021; Lesturgie et al., 2022; Rodríguez et al., 2018). This emphasizes the necessity to couple complex meta‐population models and *unstructured* models when uncovering the demographic history of a species.

Here we investigated the evolutionary history of the gray reef shark *Carcharhinus amblyrhynchos*, a coral reef‐associated shark inhabiting the tropical Indo‐Pacific. While *C. amblyrhynchos* is considered one of the most abundant reef sharks in the Indo‐Pacific, it is listed as Endangered on the IUCN red list of threatened species. With a mean size of ~190 cm (Compagno, 2001), *C. amblyrhynchos* inhabits either fringing or barrier reefs and displays patterns of reef fidelity (Barnett et al., 2012; Espinoza et al., 2014), as well as philopatry (Field et al., 2011). Tagging studies have indicated long‐range movement up to ~900 km (Barnett et al., 2012; Bonnin et al., 2019), which raise questions about the extent of residency patterns for this species. Previous molecular studies using both microsatellites and Rad sequencing did not find signatures of genetic structure at a low geographic scale such as the Great Barrier Reef (Momigliano et al., 2015, 2017), eastern Australia and Indonesia (Boussarie et al., 2022) and the Phoenix Islands archipelago (Boissin et al., 2019). Conversely, isolation by distance patterns have been found at a larger scale, and some evidence suggests that coastal abundance of reef can fuel genetic exchanges, while oceanic expanses are barriers to gene flow (Boissin et al., 2019; Boussarie et al., 2022; Momigliano et al., 2017).

**FIGURE 1:**
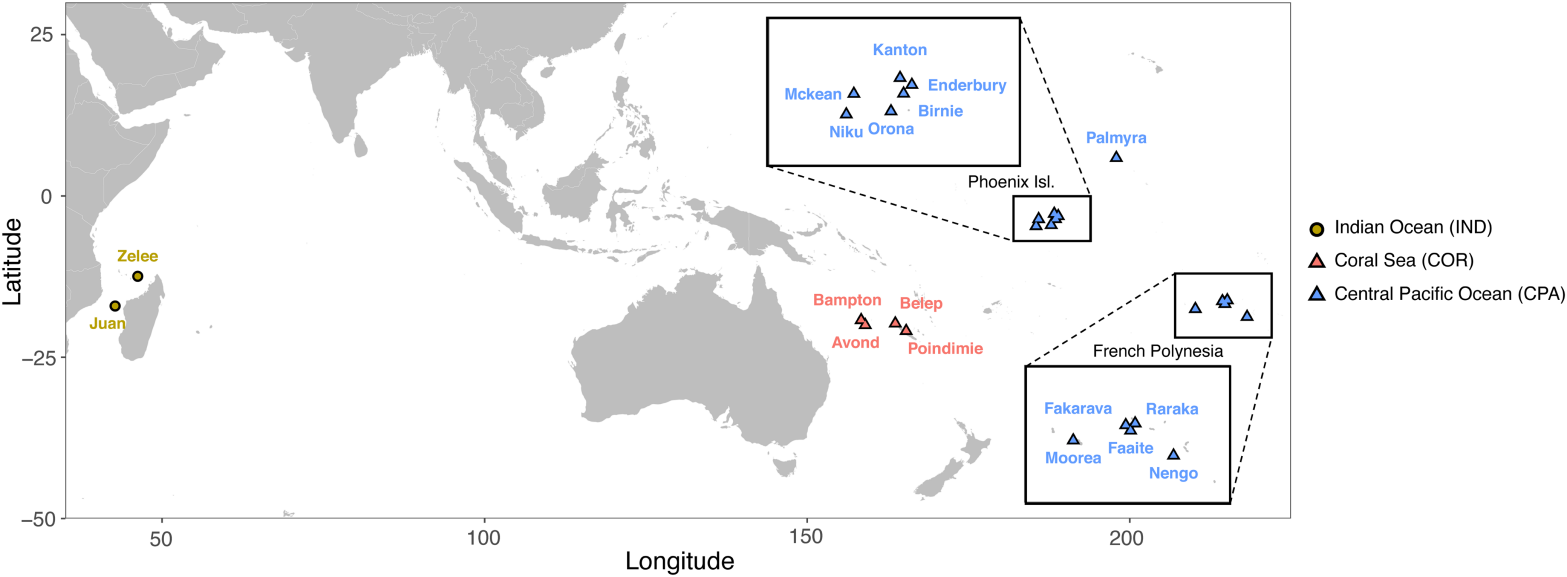
Map of the sampling sites. From west to east, Indian Ocean (IND): Juan (*n* = 13) and Zelee (*n* = 6); Chesterfield islands (CHE): Bampton (*n* = 10) and Avond (*n* = 5), New Caledonia (NCA): Belep (*n* = 7) and Poindimie (*n* = 5); Phoenix islands (PHO): Niku (*n* = 21), Mckean (*n* = 7), Orona (*n* = 11), Kanton (*n* = 10), Birnie (*n* = 2), and Enderbury (*n* = 13); Palmyra (PAL, *n* = 38); French Polynesia (POL): Moorea (*n* = 5), Fakarava (*n* = 17), Faaite (*n* = 1), Raraka (*n* = 1), and Nengo (*n* = 1). Colors represent the region of origin of the sampling sites: Indian Ocean (IND, yellow), Coral Sea (COR, red), and Central Pacific Ocean (CPA, blue).

To shed light on these contrasting findings, we sequenced DNA from 203 individuals of *C. amblyrhynchos* sampled at 18 sites from the eastern Indian Ocean to French Polynesia (Figure 1) following a double digest restriction site associated DNA protocol (dd‐RADseq, Peterson et al., 2012). The large panel of assembled loci was used to: (i) detect the occurrence and origin location of a range expansion (RE); (ii) investigate its demographic history by implementing both meta‐population and *unstructured* models; and (iii) reassess the population structure of the gray reef shark in the Indo‐Pacific. We finally compared the results here obtained with those previously found in the blacktip reef shark (*Carcharhinus melanopterus*; Maisano Delser et al., 2016; Maisano Delser et al., 2019). The two species share a very similar distribution in the Indo‐Pacific but are characterized by different habitat preferences and life‐history traits, providing an excellent opportunity to improve our knowledge on the biology of sharks.

## 2 MATERIALS AND METHODS

### 2.1 Sampling and rad sequencing

We collected 203 samples of *C. amblyrhynchos* that covered most of its longitudinal distribution range (Figure 1), with two sampling sites in the Mozambique Channel in the western Indian Ocean (IND—Juan de Nova and Zélée bank) and 16 in the Pacific Ocean (PAC). Among the PAC sampling sites, four were chosen in the Coral Sea (COR): two in the Chesterfield Islands (Bampton and Avond) and two in New Caledonia (Belep and Poindimie). The remaining samples came from the Central and Easter Pacific (CPA): six in the Phoenix Islands (Enderbury, Kanton, McKean, Niku, Orona, and Birnie), one in Palmyra Island, and five in French Polynesia (Fakarava, Moorea, Faaite, Raraga, and Nengo; Figure 1, Table 1). Total genomic DNA has been extracted and conserved in 96% ethanol using QIAGEN DNeasy Blood and Tissue purification kit (Qiagen) according to the manufacturer's protocols. We followed the double digest restriction site associated DNA (dd‐RADseq) protocol of (Peterson et al., 2012) to create a genomic library, using EcoRI and MSFI as restriction enzymes. We selected fragments of ~400 bp length and sequenced with Illumina HiSeq 2500 machine (single‐end, 125 bp).

**TABLE 1:** Summary statistics

Region	Group	Sampling site	*n*	*n*_loci_	*n*_SNP_	θ_π_ ^a^	θ_w_ ^a^	T*D* ^‡^
IND	IND	Juan	13	95,027	45,635	1.18	1.09	**0.32**
		Zelee	6	146,858	62,674	1.30	1.23	**0.26**
COR^b^	CHE	Bampton	10	89,958	82,869	2.14	2.26	**−0.22**
		Avond	5	125,710	87,817	2.10	2.15	**−0.12**
	NCA	Belep	7	120,038	103,258	2.30	2.35	**−0.11**
		Poindimie	5	107,464	72,995	2.07	2.09	**−0.05**
CPA^b^	PHO	Niku	21	49,922	53,349	2.02	2.16	**−0.25**
		McKean	7	112,711	88,258	2.13	2.14	−0.01
		Orona	11	81,725	75,423	2.15	2.20	**−0.09**
		Kanton	10	99,720	87,202	2.12	2.14	**−0.05**
		Birnie^c^	2	‐	‐	‐	‐	**‐**
		Enderbury	13	76,314	72,221	2.09	2.16	**−0.12**
	PAL	Palmyra	38	35,594	36,982	1.66	1.84	**−0.35**
	POL	Moorea	5	104,050	68,380	2.03	2.02	0.02
		Fakarava	17	71,715	66,559	2.01	1.97	**0.08**
		Faaite^c^	1	‐	‐	‐	‐	**‐**
		Raraka^c^	1	‐	‐	‐	‐	**‐**
		Nengo^c^	1	‐	‐	‐	‐	**‐**

*Note*: Sample size (*n*), total number of loci (monomorphic included) (*n*_loci_) and SNPs (*n*_SNP_), mean pairwise difference (θ_π_), Watterson theta (θ_w_), Tajima's *D* (T*D*) for all sampling sites (ranged from west to east).

^a^ Mean pairwise difference and Watterson theta are expressed per site and are multiplied by a 10^3^ factor.

^b^ COR and CPA regions are from the Pacific Ocean (PAC).

^c^ Summary statistics were not computed in sampling sites with *n* < 5.

^‡^ Tajima's *D* values in bold are significant (*p* < .001).

In the absence of a reference genome, we assembled loci de novo using *Stacks* v.2.5 (Rochette et al., 2019). Briefly, we demultiplexed the reads through the *process_radtags.pl* script and assembled the loci using the *denovo_map.pl* pipeline with the parameters *m* = 3 (minimum read depth to create a stack), *M* = 3 (number of mismatches allowed between loci within individuals), and *n* = 3 (number of mismatches allowed between loci within catalogue). We found a mean depth of coverage (over individuals and loci) of ~10× (see Section 3). Previous work suggested that such low‐coverage value may bias a correct genotype calling under the algorithm implemented in *Stacks* v.1, *Stacks* v.2, and *PyRAD* by skewing the site frequency spectrum (SFS) towards an excess of low‐frequency variants (S. Mona, P. Lesturgie, A. Benazzo, G. Bertorelle, unpublished data; see Supporting Information for details). For this reason, we followed two different bioinformatics pipelines: the first to obtain a dataset to perform analyses based on the SFS (genetic diversity, range expansion, and historical demographic inferences) and the second to investigate population structure, for which low‐frequency variants are not informative and are removed before the downstream analyses.

### 2.2 Genetic diversity

We followed the genotype‐free estimation of allele frequencies pipeline implemented in the software *ANGSD* v.0.923 (Korneliussen et al., 2014). This approach has been suggested to be more efficient for low to medium‐coverage NGS data than SNP calling algorithms (Korneliussen et al., 2014). *ANGSD* requires a reference sequence to work. To this end, we followed the framework proposed by Khimoun et al. (2020) and Heller et al. (2021), which we applied to each sampling site separately to maximize the number of loci: (i) We assembled Rad loci present in at least 80% of the sampled individuals using *Stacks* with the same parameters as above (i.e., *m* = *M* = *n* = 3); (ii) we concatenated the consensus sequences for each locus, to which we added a stretch of 120 “*N*” in order to facilitate mapping, to create an artificial reference sequence; (iii) we mapped raw reads from individual *fastq* files using the *bwa‐mem* algorithm with default parameters (Li & Durbin, 2009) against the artificial reference sequence. Using *ANGSD* filters, we discarded (i) sites with a coverage <3 (using the flag *‐minIndDepth 3*) (ii) poor quality and misaligned reads (with default parameters and flags *‐minQ20* and *‐minMapQ 20*), (iii) poor quality bases (with default parameters and flags *‐baq 1* and *‐C 50*). We further removed the last 5 bp of each locus, SNPs heterozygous in at least 80% of individuals, and loci with more than five SNPs. We finally filtered all missing data by applying the *‐minInd* filter equal to the total number of individuals present in each sampling site (Table 1). We then created a *site allele frequency likelihood (saf)* file by using the SAMtools genotype likelihood computation method with the *‐GL = 1* flag (Li & Durbin, 2009) and finally computed the folded *site frequency spectrum* (SFS) from the *saf* files using the *RealSFS* program implemented in *ANGSD*. We computed the mean pairwise difference (θ_π_), the number of segregating sites (Watterson's Theta, θ_w_), and Tajima's D (*TD*) directly from the SFS. θ_π_ and θ_w_ were standardized per site (i.e., by taking into account both monomorphic and polymorphic loci), and the significance of *TD* was evaluated under 1000 coalescent simulations of a constant population model with size θ_π_.

### 2.3 Range expansion

Genetic diversity, here measured in each sampling site as θ_π_, is expected to decay as a function of the distance from the origin of the range expansion (Ramachandran et al., 2005). Geographic distances were computed in order to take into account the ecological features as they may better represent the capacity of individuals to move between two points than linear distances. To that end, we constructed a raster of 67,894 cells using the R package *raster* (Hijmans, 2020) where each cell corresponds either to land, open sea, seamount, or reef habitat. Permeability coefficients were fixed, respectively, to 0 and 1 for land and open sea, whereas coefficients for coral reefs and seamounts were varied between 1 and 100. We applied two constraints: Coral reefs should always have the maximum relative permeability value (since they represent the only habitat for *C. amblyrhynchos*) and seamounts have permeability bounded within 1 and coral reefs' value. The most likely values were searched using a custom R script by maximizing the correlation between the geographic and genetic distances between the sampled sites. Geographic distances were computed with the *gdistance* R package under the *Least Cost* (LC) criterion algorithm (van Etten, 2017) and genetic distances were measured by the *F*_ST_ (see below). After this step, we considered each marine cell of the raster to be a potential source of origin of the range expansion (RE) and computed its distance from the sampled sites under the LC criterion with the most likely permeability values previously estimated. We correlated these distances with the genetic diversity of each sampling site to identify areas with more negative values, which are likely associated with the origin of the RE (Ramachandran et al., 2005). We limited these analyses to the PAC sites to avoid possible bias due to the gap in our sampling distribution (i.e., the lack of samples between IND and the westernmost PAC site). Nevertheless, we verified the robustness of our results to the inclusion of IND sites.

### 2.4 Historical demographic inferences

**FIGURE 2:**
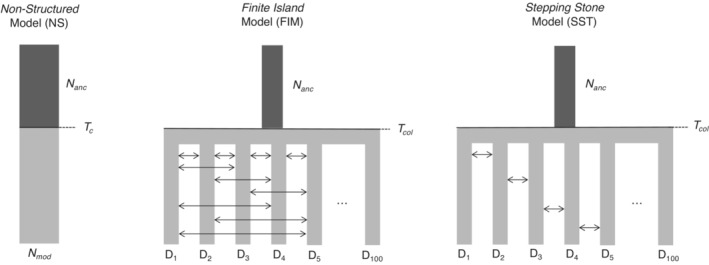
Demographic scenarios investigated in all populations with *n* ≥ 7 through an approximate Bayesian computation (ABC) framework. *N*_anc_, Ancestral effective population size; *T*_*c*_, Time of effective population size change (NS only); *N*_mod_, Modern effective population size (NS only); *T*_col_, Colonization time of the array of demes (FIM and SST); *D*_1‐100_: Demes (FIM and SST). Arrows represent the migrants exchanged (*Nm*) between demes. Details on each scenario are presented in the main text.

To account and test for meta‐population structure, we performed model selection and parameter estimation using an Approximate Bayesian Computation (ABC) framework (Bertorelle et al., 2010). We tested three demographic scenarios (Figure 2) for each sampling site, namely NS, FIM, and SST. *Model NS (no structure)*: Going backward in time, NS represents a panmictic population where the effective population size switches instantaneously at *T*_*c*_ generations from *N*_mod_ to *N*_anc_. *Model FIM (Finite Island Model)*: FIM represents a meta‐population composed of a two‐dimensional array of 10 × 10 demes (*D*_*i*_), each of the same size *N* that exchanges *Nm* migrants with any other deme each generation. Going backward in time all demes merge into a single population of size *N*_anc_ at *T*_col_ generations. *Model SST (Stepping STone)*: SST is similar to FIM, but demes exchange migrants only with their four closest neighbors. We performed 50,000 simulations under each scenario and for each sampling site independently using *fastsimcoal2* (Excoffier & Foll, 2011). We run the model selection with the Random Forest classification method implemented in the package *abcRF* (Pudlo et al., 2016) using the SFS, θ_π_, θ_w_ and *TD* as summary statistics, to which we added the first two components of the Linear Discriminant Analysis performed on the previous summary statistics as suggested by Pudlo et al. (2016) to increase accuracy. We performed 50,000 additional simulations under the most supported scenario in order to estimate the demographic parameters using the *abcRF* regression method (Raynal et al., 2019) with the same summary statistics as for the model selection. For all analyses, we performed the estimation twice to check for the consistency of the inferences. The number of trees was chosen by checking the out‐of‐bag error rate (OOB), and cross‐validation was performed for both parameter inference and model selection (hereafter, the confusion matrix) procedures. We finally modeled the variation of effective population size (*N*_*e*_) through time in each sampling site with the *stairwayplot* (Liu & Fu, 2015). The *stairwayplot* assumes that the sampled lineages come from an isolated (panmictic) population (i.e., *unstructured*), which is not true in our case (see Section 3). However, this method allows a powerful investigation of the underlying gene genealogy, which provides useful elements for interpreting the evolutionary history of a meta‐population (Lesturgie et al., 2022). All demographic inferences were performed using a generation time of 10 years and a mutation rate of 1.93e‐8 per generation and per site following Lesturgie et al. (2022).

### 2.5 Population structure

Population structure inferences were performed on the dataset obtained following the assembly pipeline implemented in *Stacks 2.5* as described above. After the *de novo* assembly step, the *population* script was called to keep loci present in at least 80% of the individuals per sampling site (*r* = .8) and with a *minor allele frequency* of 0.05, hence removing low‐frequency variants. We finally retained one random SNP per locus. Using a custom R script, we further filtered: (i) SNPs heterozygotes in more than 80% of the sample; (ii) loci with coverage higher than ~30× (which corresponds to the mean coverage plus twice the standard deviation); (iii) SNPs in the last 5 bp of the assembled locus; and (iv) loci containing more than five SNPs, after visual inspection of the distribution of segregating sites per locus. The resulting dataset was used for the following analyses. (i) *sNMF* implemented in the R package *LEA* (Frichot & François, 2015): We investigated the number of ancestral clusters *K* by running the algorithm 10 times, with values of *K* ranging from 1 to 8. We chose the most likely *K* using the cross‐entropy criterion and displayed the admixture coefficients under the best run. (ii) *DAPC* implemented in the R package *Adegenet* (Jombart et al., 2010): We varied *K* from 1 to 8 and chose the best values based on the BIC criterion. Linear discriminant functions were used to test whether individuals were correctly re‐assigned to the inferred clusters. (iii) *F*_ST_: We computed overall and pairwise *F*_ST_ between sampling sites with more than five individuals using the *PopGenome* (flag *nucleotide.F_ST*) library in R (Pfeifer et al., 2014) and tested its significance with 1000 permutations using a custom R script. Isolation by distance (IBD) was computed with a Mantel test (Mantel, 1967) between the genetic (*F*_ST_/(1−*F*_ST_)) and the geographic or LC distance matrices and tested by 1000 permutations with the *ade4* R package (Thioulouse & Dray, 2007). The Mantel test, similarly as before, was limited to PAC sites. To check for IBD in the Indian Ocean, we fit a linear model to the pairwise *F*_ST_ values computed between the PAC and IND sites and their respective geographic distances.

## 3 RESULTS

### 3.1 Genetic diversity

We discarded 30 individuals based on an excess of missing data after an initial de novo assembly. We found a mean depth of coverage of 10.77× (s.d. = 2.32) for the whole dataset. Summary statistics for all sampling sites are displayed in Table 1. The number of loci (monomorphic included) and SNPs with no missing data ranged from 35,594 to 146,858 and from 36,982 to 103,258, respectively, across sampling sites (Table 1). Genetic diversity (θ_π_ and θ_w_) was lower in IND sampling sites than in PAC (Table 1). Tajima's *D* values were positive in IND sampling sites and in Fakarava, suggesting an excess of high‐frequency variants when compared to the standard neutral model. Conversely, we found negative and significant Tajima's *D* values in all other PAC locations (except for Moorea and Mckean), suggesting an excess of low‐frequency variants compared with the standard neutral model (Table 1).

### 3.2 Range expansion

**FIGURE 3:**
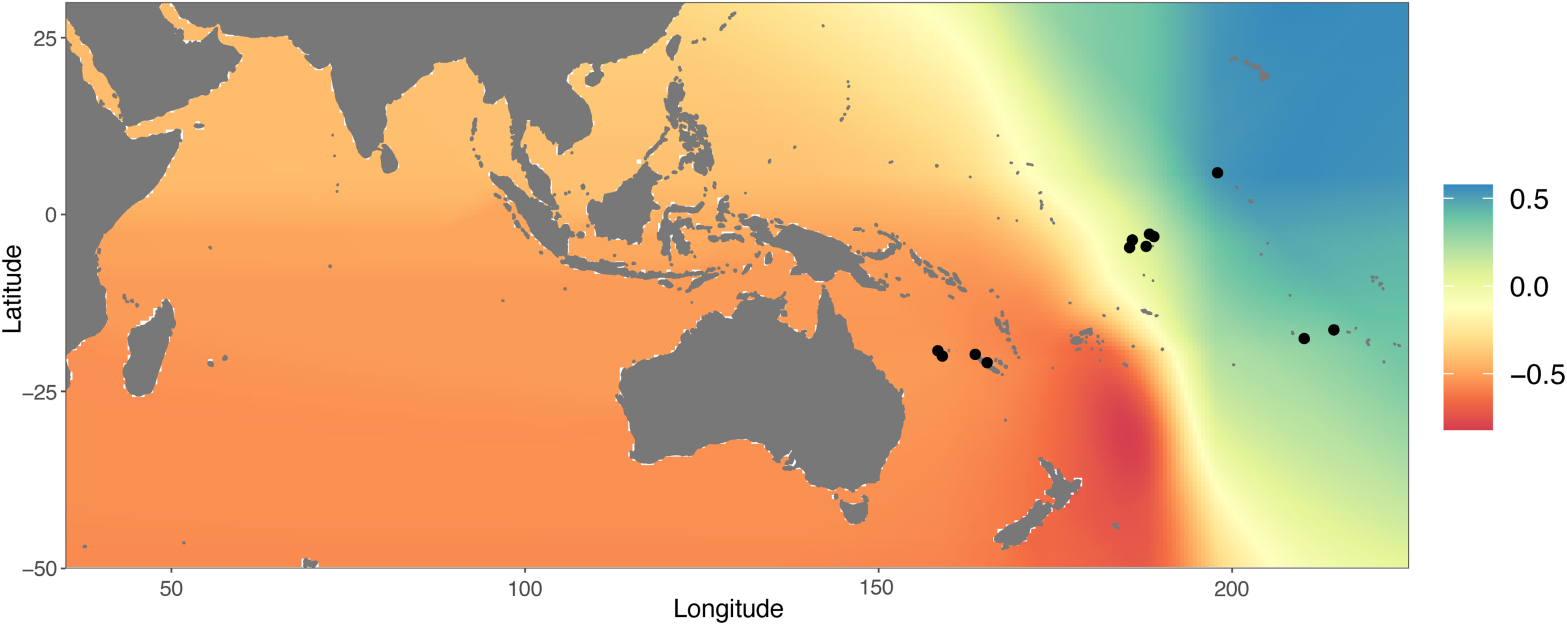
Correlation map between genetic diversity (θ_π_) and least cost (LC) distances when considering Pacific Ocean sampling sites only. Each cell is colored according to the correlation coefficient value computed between θ_π_ and the LC distance from the putative origin of the range expansion (RE). Black dots represent the sampling sites considered.

The permeability coefficients maximizing the correlation between genetic and the LC distances were very similar between the three habitat types. Indeed, we estimated the values of 1:1.02:1.02 for open sea, coral reef habitat, and seamounts, respectively. These values were retained for the following RE and IBD analyses. We plotted the correlation map computed using PAC sites only in Figure 3. The most negative correlation coefficients are concentrated close to the COR sampling sites, suggesting that the most likely origin of the RE is slightly east to the IAA region (Figure 3). We found consistent results when adding IND sites to the analysis (Figure S1), despite the geographic unbalanced distribution of our samples.

### 3.3 Historical demographic inferences

We investigated the demographic history for all sampling sites with *n* ≥ 7. We first used an ABCRF framework to compare demographic scenarios (Figure 2). SST was the most supported scenario in all locations, with posterior probabilities ranging from 0.48 to 0.78 and similar classification error rates among locations (Tables 2 and S1). The median *Nm* ranged from ~6 to ~14 (Table 2). Posterior distributions of *Nm* were overlapping and clearly distinct from the prior distribution (Figure S2), and both the squared mean error (SME) and the mean root squared error (MRSE) were small among locations, suggesting reliable estimates (Table S2). Posterior distributions of *T*_col_ overlapped among locations (Figure S2). Juan de Nova displayed a lower *N*_anc_ median value (~21 k) than PAC sampling sites (ranging from ~34 k to ~50 k) although all credible intervals overlapped (Figure S2 and Table 2). Surprisingly, the ABC estimates of *T*_col_ and *N*_anc_ for the Mckean sampling site were inconsistent with any other PHO sampling sites (Figure S2 and Table 2). However, both SME and the MRSE for these two parameters were generally one order of magnitude larger than those estimated for *Nm* in all sampling sites (Table S2), suggesting less accurate estimates for *T*_col_ and *N*_anc_.

**TABLE 2:** ABC estimation. Posterior probability (PP) of the stepping‐stone model (SST) and its parameters (median value and 95% credible interval in parentheses).

Region	Group	Sampling site	PP	*Nm*	*T*_col_	*N*_anc_
IND	IND	Juan	0.67	5.7 (1.77–17.72)	257,800 (8086–658,471)	21,086 (399–52,652)
COR^a^	CHE	Bampton	0.73	11.41 (3.97–19.03)	188,782 (127,761–577,503)	45,965 (27,556–49,856)
	NCA	Belep	0.51	7.8 (2.84–20.82)	241,218 (112,840–843,171)	49,239 (7346–56,316)
CPA^a^	PHO	Enderbury	0.65	8.36 (2.9–20.9)	197,070 (95,260–678,828)	43,602 (14,665–51,030)
		Kanton	0.7	8.16 (2.84–16.55)	257,718 (118,094–789,320)	41,236 (2534–52,613)
		McKean	0.6	7.09 (2.98–15.25)	621,535 (158,650–836,223)	18,881 (4968–51,387)
		Niku	0.59	14.1 (3–30.55)	152,035 (66928–598,129)	43,495 (9184–48,625)
		Orona	0.48	7.7 (2.93–15.31)	269,621 (137,304–799,518)	41,680 (4575–51,152)
	PAL	Palmyra	0.73	13.39 (4.16–27.22)	142,756 (62,402–445,380)	32,542 (9502–37,524)
	POL	Fakarava	0.72	10.2 (2.68–15.34)	256,744 (110,875–780,150)	40,502 (3091–49,533)
			Priors	^b^ *U* [0.0001; 100]	*U* [100; 1,500,000]	*U* [100; 100,000]

^a^ COR and CPA regions are from the Pacific Ocean (PAC).

^b^ The prior distribution of *Nm* is the product of two uniforms (one for *N* and one for *m*).

**FIGURE 4:**
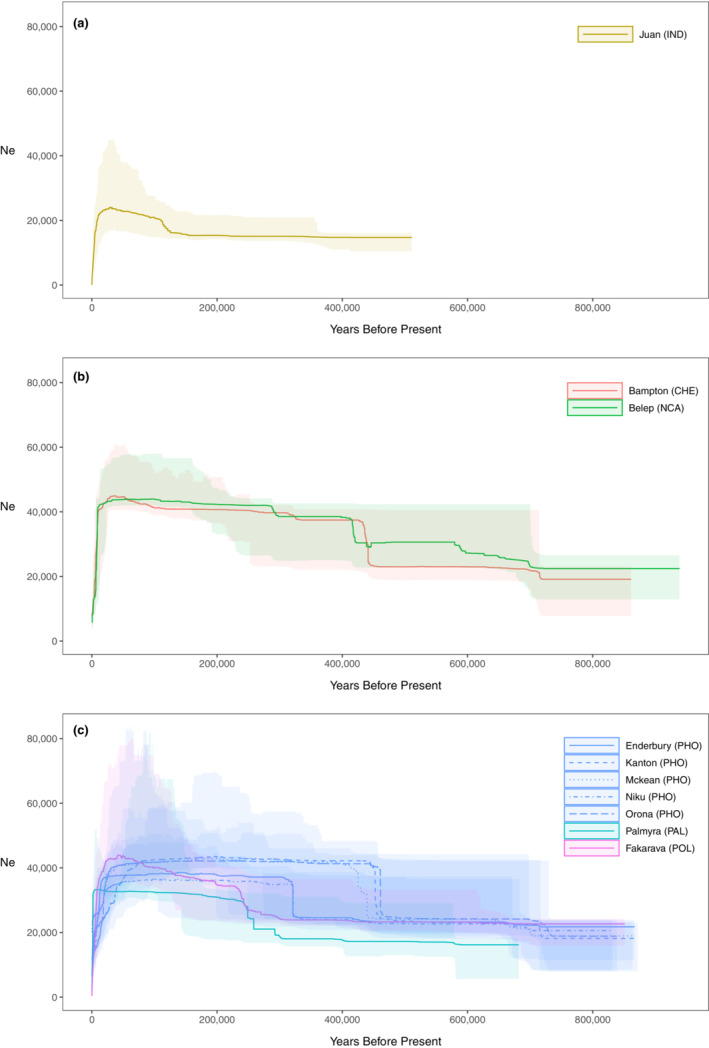
Variation of the effective population size (*Ne*) through time and its 75% confidence interval estimated by the *stairwayplot* for sampling sites of *n* ≥ 7 in IND (a), COR (b), and CPA (c) regions

We further investigated the variation of *Ne* through time using the *stairwayplot* algorithm (Figure 4). We detected a broadly similar *Ne* dynamic across sampling sites that we summarized for simplicity in three time periods: Looking forward in time, we observed an ancestral expansion followed by a constant phase and a final systematic strong decrease in recent times (Figure 4). However, we found three main differences between IND and PAC sampling sites: (i) The expansion time was around twice as recent in IND than in PAC (~180ky B.P. vs. ~400ky B.P); (ii) the strength of the expansion is much stronger in PAC sampling sites; (iii) *Ne* during the constant period reached a value of ~40,000 in PAC sampling sites and of only ~20,000 in IND, consistent with the computed θ (Table 1). The PAC sampling sites showed a remarkably homogeneous *stairwayplot*, with only the peripheral sites (Fakarava and Palmyra) having a slightly more recent ancestral expansion (Figure 4).

### 3.4 Population structure

**FIGURE 5:**
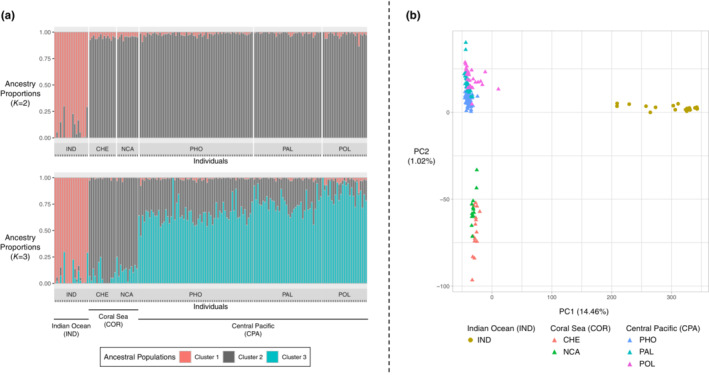
Individual‐based population structure analyses. Ancestry proportions retrieved using the *sNMF* algorithm with *K* = 2 and *K* = 3 ancestral populations (a) and principal component analysis (b)

After filtering, 88,276 variable loci were retained to perform individual‐based structure analyses. Both *sNMF* and the *DAPC* clustering algorithms found *K* = 2 as the most likely number of ancestral populations or clusters (Figures S3 and S4a). The ancestral populations inferred by *sNMF* perfectly matched the two oceanic regions, namely the Indian and the Pacific Ocean: The ancestry proportion of *cluster 1* in IND samples ranged from 70% to 100% while the ancestry proportion of *cluster 2* in PAC samples ranged from 87% to 100% (Figure 5a). This highlights slightly more admixture in IND than in PAC samples. We retained one LD function in the *DAPC*, which correctly re‐assigned all individuals from IND and PAC to *cluster 1* and *cluster 2*, respectively (Figure S4b). We further investigated *K* = 3 under both algorithms and found three main results: (i) The ancestral populations or clusters clearly identify three geographic areas corresponding to IND, COR, and CPA regions (Figures 5a and S5); (ii) the ancestry proportion of *cluster 3* follows a clinal distribution, steadily increasing in frequency from West (Indian Ocean) to East (French Polynesia; Figure 5a); (iii) all individuals belonging to the three areas are correctly re‐assigned to the three clusters by the *DAPC* computed with two LD functions (Figure S4b). We then computed a PCA, which showed similar results, with the first principal component explaining ~14.5% of the total variance and clearly separating individuals coming from the two oceans (Figure 5b). The second axis segregated CPA from COR samples. In agreement with the cluster analyses, CPA and COR are only slightly differentiated as the second principal component explains only ~1% of the total variance. The second axis also suggested a clinal differentiation between the two clusters (Figure 5b).

**FIGURE 6:**
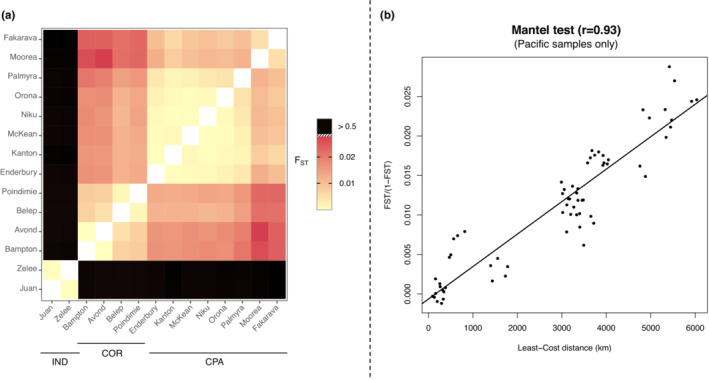
Population‐based population structure analyses computed with populations of *n* ≥ 5. Heat map representing the pairwise *F*_ST_ values between sampling sites (a) and isolation by distance (IBD) plot considering Pacific sampling sites only (b)

Population‐based analyses were performed on a reduced dataset excluding sampling sites with less than *n* = 5 individuals. We therefore retained 14 sampling sites, *n* = 168 individuals, and 88,824 variable loci and obtained an overall *F*_ST_ = 0.25 (*p*‐value < .001). The pairwise *F*_ST_ highlighted a strong differentiation between Indian and Pacific sampling sites with values ranging from 0.53 to 0.56 (and always significant, *p*‐value ≤ .001, Table S3). By contrast, comparisons within oceanic regions never exceed 0.03 (Figure 6a) with values not always statistically significant. Consistently with clustering results, a heatmap displaying pairwise *F*_ST_ values visually suggests the existence of the three clusters previously identified (Figure 6a). However, the average differentiation between COR and CPA is only slightly higher than within‐group comparisons (Figure 6a). Moreover, we found a strong signature of isolation by distance (IBD) within the Pacific Ocean (using PAC sites only), since the correlation between the *F*_ST_ and geographic or LC distance matrices was high and significant (Mantel test: *r* = .93; *p*‐value < .001 in both cases, Figure 6b). The correlation between genetic and geographic distances by considering only IND versus PAC pairwise distances was also considerable although lower than in PAC region only (*r* = .77, Figure S5).

## 4 DISCUSSION

### 4.1 Range expansion

Range expansions (RE) occur by a series of founder effects leading to the fixation of novel alleles and the decay in genetic diversity as colonization progresses (Excoffier et al., 2009). They also leave specific signatures in the gene genealogy of lineages sampled from a deme of the meta‐population (Maisano Delser et al., 2016; Ray et al., 2003) and in the extent of population structure (Mona, 2017; Mona et al., 2014). Testing for the occurrence of a RE is therefore fundamental to understanding the evolutionary history of a species. Here, the spatial distribution of genetic diversity suggested the occurrence of a RE most likely starting east of the Indo‐Australian Archipelago (IAA). The inferred origin area was large (Figure 3), likely due to low differences in θ_π_ between Pacific sampling sites (Table 1) but robust to the inclusion of samples from the Indian Ocean (Figure S1). The scenario of a RE was corroborated by other evidence. First, the strong and significant correlation coefficient between genetic and geographic distances in the Pacific Ocean (*r* = .93; Mantel *p*‐value < .001, Figures 6b and S5). This result alone would not be conclusive, since a similar pattern is also expected under equilibrium isolation by distance, but it strengthens our previous findings. Second, the historical demography inferences performed in each sampled deme showed that the pattern of genetic variability was most likely the outcome of a nonequilibrium meta‐population structured according to a stepping‐stone migration matrix (Table 2). In this context, both the colonization times of the meta‐population estimated by the ABC (Figure S2) and the expansion times retrieved by the *stairwayplot* (Figure 4) harbor the signature of the RE process (Lesturgie et al., 2022): The oldest times are expected to be close to the centre of origin of the RE, while the more recent ones are likely associated to the edge of the colonization wave(s). While the large variance in *T*_col_ estimated by ABC does not allow for an accurate interpretation of the temporal dynamics of colonization through the Indo‐Pacific, the expansion times highlighted by the *stairwayplot* are consistent with the RE scenario. Indeed, all sampling sites display a simultaneous expansion time around ~400 ky B.P. (Figure 4) except for Palmyra, Fakarava, and Juan de Nova, which are the sites, respectively, further east (Palmyra and Fakarava) and west (Juan de Nova) to the inferred origin of the RE. In summary, all the evidence presented thus far points to an origin of *C. amblyrhynchos* east of IAA (particularly, east of New Caledonia), from which two migration waves took place, one to the East Pacific and the other to the Indian Ocean, with the Mozambique Channel being probably one of the last areas to have been colonized.

Our hypothesis is in line with the recent results of Walsh et al. (2022), but they detected the origin of the RE within rather than eastward the IAA, using a similar genetic diversity decay approach. This discrepancy may be mostly due to the sensibility of this algorithm to the spatial distribution of the sampled populations (Peter & Slatkin, 2013), which differs considerably between the two studies. Another source of discrepancy may lie in the different bioinformatics pipelines. Walsh et al. (2022) assembled loci with *PyRAD* (Eaton, 2014), whose calling algorithm requires high coverage data to correctly identify genotypes (Rochette et al., 2019). Here, we used the genotype‐free approaches implemented in *ANGSD* to avoid possible skew towards low‐frequency variants in Radseq experiment with low to medium coverage (Heller et al., 2021; S. Mona, P. Lesturgie, A. Benazzo, G. Bertorelle, unpublished data). To shed more light on this issue, we carefully compared our results (obtained with *ANGSD*) to those obtained by three assembly and calling pipelines (namely, *PyRAD* (Eaton, 2014), *Stacks* v.1.48 (Catchen et al., 2013) and *Stacks* v.2.5 (Rochette et al., 2019), see Supplementary Methods) using the Bampton sampling site as a test case. All three SFS displayed an excess of singletons in comparison to the one inferred by *ANGSD* (Figure S6b), clearly determining not only a stronger ancestral expansion but also the absence of the recent bottleneck when fed to the *stairwayplot* algorithm (Figure S6a). These results are consistent with Heller et al. (2021), as we found an excess of low‐frequency variants when using the *Stacks* pipeline compared with the genotype likelihood approach implemented in *ANGSD*. Consequently, we highlight that the SFS reported by Walsh et al. (2022) could be slightly biased towards an excess of low‐frequency variants.

The RE scenario, characterized by a centre of origin and two independent colonization waves, is similar to the one inferred for *C. melanopterus* by Maisano Delser et al. (2019), a species whose range distribution overlaps with that of the gray reef shark. However, the most likely origin of the RE was located within the IAA for *C. melanopterus*, a well‐known centre of origin for many teleost fishes (Cowman & Bellwood, 2013), and a biodiversity hotspot (Allen, 2008). The difference observed between *C. amblyrhynchos* and *C. melanopterus* could result from the more balanced sampling scheme of Maisano Delser et al. (2019), who could cover more homogeneously the Indo‐Pacific. More samples from the IAA will be needed to refine our estimates. More generally, it will be interesting in the next future to explicitly investigate the role of the IAA for coral reef biodiversity fauna and to reconstruct the colonisations routes in the Indo‐Pacific, using population genetics modeling applied to genomics data on multiple marine species to extract more general patterns (see for example Delrieu‐Trottin et al., 2020).

### 4.2 Historical demography

The ABC framework not only provided another evidence in favor of a nonequilibrium meta‐population scenario through the model selection analysis but also allowed us to further refine our understanding of the evolutionary history of the gray reef shark. By analyzing each deme separately, we found an overlapping posterior distribution of *Nm* with an average mode of ~10 (Table 2 and Figure S2). *C. amblyrhynchos*, similar to *C. melanopterus*, is strongly dependent on reefs, whose distribution is not homogenous in the Indo‐Pacific (Figure S7). We would have expected the connectivity in each sampled deme to be highly correlated to the distribution of coral reefs in its neighborhood, as it was previously observed in *C. melanopterus* (Maisano Delser et al., 2019). However, the two species differ in their dispersal behaviors: While gray reef sharks perform long‐distance movements of at least ~900 km (Barnett et al., 2012; Bonnin et al., 2019; White et al., 2017), the blacktip reef shark exhibits a range of movement not exceeding ~50 km (Mourier & Planes, 2013). Our results reinforce the idea that the neighborhood size in the two species is very different, with *C. amblyrhynchos* being able to cross expanses of open ocean and therefore being less sensitive to coral reef concentration than *C. melanopterus*.

The homogeneity in the signature of genetic variation in each deme was confirmed by the *stairwayplot* analyses (Figure 4), contrasting with the heterogeneity previously described for *C. melanopterus* (Maisano Delser et al., 2019). All demes showed an ancestral expansion followed by a period of stasis and a strong bottleneck in recent times. We recently showed that these three time periods are the typical signature of the variation in the coalescence rate through time due to the meta‐population structure, with the slight differences observed between sites being only due to their specific colonization time (Lesturgie et al., 2022). This result confirms the similarity of dispersal patterns throughout the Indo‐Pacific. Similarly, the signature of bottleneck observed in recent times for all demes (Figure 4) is also the expected consequence of population structure (Chikhi et al., 2018; Lesturgie et al., 2022; Mazet et al., 2015; Rodríguez et al., 2018). This is true even when explicitly modeling spatial expansion with low *Nm* and colonization time of the same order as the one estimated in the gray reef sharks (as shown by the TD distribution, Mona, 2017). Unfortunately, population structure and demographic decline affect the SFS in a similar fashion making it impossible to quantitatively disentangle the contribution of both to the observed bottleneck estimated using RADseq data (Lesturgie et al., 2022). We stress that investigating local recent changes in connectivity or demographic events will clearly require whole genome sequencing coupled with inferential methods based on the IICR (Arredondo et al., 2021) and/or linkage disequilibrium (Boitard et al., 2016). More generally, the next challenge will be to perform a full modeling of species structured in many demes as the gray reef shark. Here we took a simplified approach by considering each sampling site separately and by modeling the unsampled demes to estimate local migration rates. We are aware that in the future more data will be needed to explore complex demographic scenarios integrating RE that include both all sampled demes and the unsampled ones.

### 4.3 Population structure

The results presented so far suggest that the dispersal abilities of *C. amblyrhynchos* are similar throughout the Indo‐Pacific and independent of the availability of coral reefs. However, this cannot exclude the presence of barriers to gene flow, which may have shaped the connectivity between demes. For widely distributed marine species, detecting such barriers may help to delineate management units and to take proper conservation measures in relation to fisheries (Dudgeon et al., 2012). Several evidence point to an absence of barriers to gene flow in the gray reef shark. First of all, we found a strong IBD pattern with a significant correlation between genetic and geographic distances of > 0.9 when considering only PAC samples (Figure 6b) and a linear relation of smaller intensity between IND and PAC samples (Figure S5). Remarkably, these values are not affected by computing geographic distances between sampling sites under an LC approach. Indeed, the permeability values maximizing the correlation are (almost) the same for the different types of habitats. This suggests that different geographic features do not affect the direction of gray reef shark migrations, indicating, albeit indirectly, the absence of barriers to dispersal, consistently with the occasional long‐distance swims detected across the open ocean (Barnett et al., 2012; Bonnin et al., 2019; White et al., 2017). When strong IBD is present, it is difficult to attribute a biological meaning to groups identified by clustering algorithms (Meirmans, 2012). Both the *sNMF* and PCA analyses suggested a strong separation between IND and PAC samples (Figure 5), with the latter subdivided into two weakly divergent clusters (Figures 5 and S8). The IND ancestral components diminished remarkably continuously eastward, once again supporting an IBD structure (Figure 5a) rather than the presence of barriers to gene flow. This is consistent with the pairwise *F*_ST_ matrix, where intra‐Pacific comparisons did not exceed ~0.03 while the inter‐oceanic comparisons have an average *F*_ST_ of ~0.54 (Figure 6a). Defining management units within the PAC seems therefore inappropriate in the case of the gray reef shark, as genetic variations are rather continuous. This contrasts with what was previously suggested by Boissin et al. (2019) on the Pacific scale; however, their results were based on a small number of microsatellites and they did not model IBD between the sampling points.

The pitfall of our study is to extrapolate the dynamic of the gray reef shark at the scale of its whole range by focusing mostly on the Pacific Ocean. Indeed, even if the species seems to follow an IBD pattern also from Chagos to Eastern Australia (Boussarie et al., 2022; Momigliano et al., 2017), the level of population differentiation appears to be higher than what we found in the Pacific for similar geographic distances. However, while the distribution of coral reefs in the Pacific Ocean is scattered due to the presence of many archipelagos, coral reefs in the Indian Ocean are more concentrated on the coastal edge of the Asian and African continents (Figure S7). The effective distance between sampling sites within the Indian Ocean would therefore be larger than in the Pacific Ocean, where coral reefs would act as stepping stones to facilitate the colonization process and further migrations. This could also account for the different linear relationship estimated in the Pacific versus the one estimated between Pacific and Indian sampling sites (Figure S5).

## 5 CONCLUSION

We explored the evolutionary history of the gray reef shark throughout most of its range in the Indo‐Pacific and contrasted the results with those previously obtained for the blacktip reef shark (Maisano Delser et al., 2019). The two species are among the most abundant reef sharks (MacNeil et al., 2020), share an almost overlapping distribution in the Indo‐Pacific, and are both strictly coral reef‐dependent species. Despite similarities in the RE dynamic, patterns of genetic diversity and population structure are very different between the two species. First, *C. melanopterus* is significantly more structured than *C. amblyrhynchos* at similar spatial distances (for comparison, *F*_ST_ values are ~30 times higher when comparing French Polynesia vs. New Caledonia, see Table S5 of Maisano Delser et al. (2019) and our Table S3). Second, *C. amblyrhynchos* shows homogeneous migration rates and demographic signals throughout its whole distribution whereas *C. melanopterus* is more sensitive to the spatial distribution of coral reefs with a connectivity largely dependent on the short scale reef availability (Maisano Delser et al., 2019). Indeed, migration rates estimated in areas with extensive coral reefs coverage (e.g., the Great Barrier Reef) are much higher compared to those estimated in isolated islands/atolls in the Indo‐Pacific (Maisano Delser et al., 2019), something that we did not observe for *C. amblyrhynchos*. All these differences can be explained by the life‐history traits related to the dispersal abilities of the two species, with *C. amblyrhynchos* moving more freely in open sea expanses compared with *C. melanopterus*, lowering the impact of coral density on the observed genetic diversity. However, it will be important in the next future to precisely characterize the extent of the neighborhood size for both species. To this end, ecological and genomic data need to be coupled: This will help to carefully decipher how many management units are necessary for species conservation and at which scale they should be established.

### 5.1 Comparison to Walsh et al., 2022 results

A reviewer raised some concerns about our claims related to the discrepancies between Walsh et al. (2022) results and ours. The reviewer first strongly stated that Walsh et al. (2022) results are not biased because of the coverage. Mean and median values reported for each individual (obtained by setting the minimum read depth assembly parameter to 6) are between 15× and 20×: We argue that this value may not be high enough to obtain unbiased results given the variant calling algorithm they use (the one implemented in Pyrad, which is the same as *Stacks* v.1: see Rochette et al. (2019) for a discussion on this topic). More generally, it has been shown that genotype‐free pipelines (such *ANGSD*, which we applied here) perform better than the direct calling approaches in Rad experiments (Warmuth & Ellegren, 2019) and that the direct calling could skew the SFS towards an increase of singletons (Heller et al., 2021). Here, we do not claim that Walsh et al. (2022) results are all biased—we simply stress that (i) their SFSs show an increase of singletons when compared to our data (this is particularly striking when comparing the Bampton sites, present in both studies); (ii) when applying their pipeline to our data (which are low coverage) we found an excess of low‐frequency variants compared with the results obtained by *ANGSD* (Figure S6b). These considerations suggest that Walsh et al. (2022) data could suffer from a slight skew to an excess of low‐frequency variants, which, in turn, would explain the detection of an ancestral expansion signal and the lack of a recent decrease of effective population size in their *stairwayplot* results (which we observed in our data, compare their Figure 3 with our Figure 4).

The Reviewer raised a second point concerning our results: If a RE occurred (as both studies suggest more or less explicitly), then we should not observe a recent bottleneck in the sampled demes. This, according to the Reviewer, would suggest that our results are biased (while Walsh et al., 2022 are correct). This claim is unjustified for two main reasons: (i) a recent bottleneck at the local or global scale and/or a decrease in connectivity would inflate SNPs with average frequency variants affecting the reconstructed *Ne* trajectory particularly in recent times in any meta‐population model (i.e., also in RE); (ii) in line with this, and more generally, the behavior of a sample of lineages from a deme depends specifically from the parameters of the RE: In other words, any possible SFS (and so the coalescence rate or *Ne* trajectory through time estimated out of it) can be obtained by varying these parameters. Similarly, an unstructured model can mimic the SFS produced under any meta‐population model simply varying the function of *Ne* variation through time (Chikhi et al., 2018; Mazet et al., 2016). Observing a deficit of low‐frequency variants in a deme is therefore not at all inconsistent with a species experiencing a RE (see Ray et al. (2003); Wegmann et al. (2006); Mona et al. (2014) and Mona (2017), among others). Moreover, the estimated time of the ancestral expansion in the gray reef shark is of the order of tens of thousands of generations and the exchanged migrants *Nm* ~ *10* per generation. Spatial explicit RE simulations already proved that under these parameters' combination *TD* can be positive (Mona, 2017) and instantaneous colonization models (lacking the spatial components) SST show signature of recent declines (Lesturgie et al., 2022) in agreement with theoretical predictions (Chikhi et al., 2010; Chikhi et al., 2018; Mazet et al., 2016; Rodríguez et al., 2018).

## AUTHOR CONTRIBUTIONS

**Pierre Lesturgie:** Conceptualization (equal); data curation (lead); formal analysis (lead); investigation (lead); methodology (lead); software (lead); validation (equal); visualization (lead); writing – original draft (equal); writing – review and editing (equal). **Camrin D. Braun:** Resources (equal); writing – review and editing (supporting). **Eric Clua:** Resources (equal); writing – review and editing (supporting). **Johann Mourier:** Resources (equal); writing – review and editing (supporting). **Simon R. Thorrold:** Resources (equal); writing – review and editing (supporting). **Thomas Vignaud:** Resources (equal); writing – review and editing (supporting). **Serge Planes:** Resources (equal); writing – review and editing (supporting). **Stefano Mona:** Conceptualization (equal); data curation (supporting); formal analysis (supporting); funding acquisition (lead); investigation (supporting); methodology (supporting); project administration (lead); resources (equal); software (supporting); supervision (lead); validation (equal); visualization (supporting); writing – original draft (equal); writing – review and editing (equal).

## Supporting information


Appendix S1.

## Data Availability

VCF files, SFS, and scripts are available from the Dryad Digital Repository: doi:10.5061/dryad.547d7wm9b. Fastq sequence files are available from the GenBank at the National Center for Biotechnology Information short‐read archive database (BioProject ID: PRJNA917473).
