# Supplementary material for: Like a rolling stone: Colonization and migration dynamics of the gray reef shark (Carcharhinus amblyrhynchos)
Source: Ecol Evol. 2023 Jan 10;13(1):e9746. doi: 10.1002/ece3.9746 (PMC9831972; doi:10.1002/ece3.9746)
Supplement: Supplementary file 1 — Appendix S1. [file ECE3-13-e9746-s001.pdf]

Supplemental Information for:

Like a rolling stone: colonization and migration dynamics of the gray reef shark (*Carcharhinus amblyrhynchos*)

Pierre Lesturgie[1], Camrin D. Braun[2], Eric Clua[3,4], Johann Mourier[3,5], Simon R. Thorrold[2], Thomas Vignaud[3], Serge Planes[3,4], Stefano Mona[1,4*]

[1] Institut de Systématique, Evolution, Biodiversité (ISYEB), Muséum National d'Histoire Naturelle, EPHE-PSL, Université PSL, CNRS, SU, UA, Paris, France

[2] Biology Department, Woods Hole Oceanographic Institution, Woods Hole, MA 02543, USA

[3] Laboratoire d'Excellence CORAIL, Papetoai, French Polynesia.

[4] EPHE, PSL Research University, Paris, France

[5] Université de Corse Pasquale Paoli, UMS 3514 Plateforme Marine Stella Mare, 20620 Biguglia, France

* Author for corresponding: Stefano Mona, Institut de Systématique, Evolution, Biodiversité (ISYEB), EPHE-PSL, Université PSL, MNHN, CNRS, SU, UA, Paris, France, stefano.mona@mnhn.fr.

Supplementary Methods

Comparison of site frequency spectrum using different assembly and variant calling pipelines

To empirically investigate the influence of low coverage on variant calling, we investigated the site frequency spectrum (SFS) reconstructed in the Bampton sampling site (N=10) using four assembly and variant calling pipelines:

(1) **S1:** This pipeline is based on *Stacks* v.1.48 (Catchen *et al.*, 2013). We implemented the same assembly parameters as those applied with *Stacks* v.2.5 and detailed in the main text (namely, m=3, n=3, and N=3). *Stacks* v.1.48 uses the calling algorithm of (Lynch, 2009) which requires high coverage data for accurate genotype inference (Rochette *et al.*, 2019). Using a custom R script, we filtered: *(i)* SNPs heterozygotes in more than 80% of the sample; *(ii)* loci with coverage higher than the mean coverage plus twice the standard deviation; *(iii)* SNPs in the last 5bp of the assembled locus; and *(iv)* loci containing more than five SNPs, after visual inspection of the distribution of segregating sites per locus.

(2) **PY:** This pipeline uses the assembly algorithm implemented in *PyRAD* (Eaton, 2014). We applied the same parameters of Walsh *et al.*, (2022), in order to compare their results to ours. The clustering threshold (level of similarity between sequences to be considered homologous) was set to 0.9, reads with more than 5 low quality bases were discarded, the minimum read depth for base calling was set to 6 and the maximum to 1000. The calling algorithm of *PyRAD* is the same as *Stacks* v.1.48. Loci with more than 5 SNP and sites with higher heterozygosity than 0.5 were also discarded using *PyRAD* pipeline. Using a custom R script, we additionally filtered depth by retaining only sites in the 90% core of the distribution of depth following Walsh *et al.*, (2022).

(3) **S2:** This pipeline is based on *Stacks* v.2.5 (Rochette *et al.*, 2019) and mainly differs from S1 and PY in the calling algorithm. *Stacks* v.2.5 implements the population-based bayesian framework of (Maruki and Lynch, 2017) for variant calling, which is supposed to be more accurate for low coverage data (Rochette *et al.*, 2019). The assembly step and filters were performed similarly to S1 above.

(4) **AN:** This pipeline is the one we used in the main text for all sampling sites. It is based on a first assembly of a pseudo-reference sequence (as in (Khimoun *et al.*, 2020; Heller *et al.*,

2021) against which raw reads are mapped back, before using *ANGSD* (Korneliussen *et al.*, 2014) for the genotype free allele frequency estimation. This pipeline has been previously described and successfully applied to low-coverage RAD-seq data (Heller *et al.*, 2021; Lesturgie, Planes, *et al.*, 2022) and it is detailed in the main text.

We retained only loci with no missing data (monomorphic loci were used to properly scale the genetic diversity). The folded SFS was then computed by using a custom R script except for the folded SFS produced through the AN pipeline which was directly inferred using the *RealSFS* program implemented within the *ANGSD* framework. We computed the normalized SFS as in (Lapierre *et al.*, 2017) to compare the distribution of alleles frequency between the four pipelines. The expectation of the normalized SFS is a horizontal line in a panmictic population of constant *Ne* (the standard coalescent model). The normalized SFS allows an immediate and qualitative description of the excess or deficit of low frequency variants compared to the standard coalescent model. We then inferred the variation of effective size (*Ne*) through time by modelling the SFS with the *stairwayplot* software (Liu and Fu, 2020). To be correctly scaled, the *stairwayplot* needs the total number of sites without missing data (monomorphic sites included) which were either directly extracted from the variant calling output (AN, S1 and S2) or estimated from the missing data rate detected in variant sites and the total number of sites assembled (PY). To compare the inferred *stairwayplot* with Walsh *et al.*, (2022), we used their same generation time of 16.4 years and mutation rate μ=1.9434e-08 per site per generation. This mutation rate was taken from (Maisano Delser *et al.*, 2016), who estimated it based on the exon capture data of the black tip reef shark *C. melanopterus*. This value was later adjusted to represent a true genomic average, since exon capture represent a genomic sample enriched in conserved regions (Lesturgie, Lainé, *et al.*, 2022; Lesturgie, Planes, *et al.*, 2022). Therefore, in the main text we used the corrected mutation rate of 1.93e-8 per site per generation and a generation time of 10 years as in (Lesturgie, Planes, *et al.*, 2022).

References

Catchen J, Hohenlohe PA, Bassham S, Amores A, Cresko WA (2013). Stacks: an analysis tool set for population genomics. *Mol Ecol* **22**: 3124–3140.

Eaton DAR (2014). PyRAD: Assembly of de novo RADseq loci for phylogenetic analyses. *Bioinformatics* **30**: 1844–1849.

Heller R, Nursyifa C, Garcia-Erill G, Salmona J, Chikhi L, Meisner J, *et al.* (2021). A reference-free approach to analyse RADseq data using standard next generation sequencing toolkits. *Mol Ecol Resour* **21**: 1085–1097.

Khimoun A, Doums C, Molet M, Kaufmann B, Peronnet R, Eyer PA, *et al.* (2020). Urbanization without isolation: The absence of genetic structure among cities and forests in the tiny acorn ant Temnothorax nylanderi. *Biol Lett* **16**.

Korneliussen TS, Albrechtsen A, Nielsen R (2014). ANGSD: Analysis of Next Generation Sequencing Data. *BMC Bioinformatics* **15**: 1–13.

Lapierre M, Lambert A, Achaz G (2017). Accuracy of Demographic Inferences from the Site Frequency Spectrum: The Case of the Yoruba Population. *Genetics* **206**: 439–449.

Lesturgie P, Lainé H, Asuwalski A, Chifflet-Belle P, Maisano Delser P, Magalon H, *et al.* (2022). Life history traits and biogeographic features shaped the complex evolutionary history of an iconic apex predator (Galeocerdo cuvier). *Res Sq*: 1–23.

Lesturgie P, Planes S, Mona S (2022). Coalescence times, life history traits and conservation concerns: An example from four coastal shark species from the Indo-Pacific. *Mol Ecol Resour* **22**: 554–566.

Liu X, Fu YX (2020). Stairway Plot 2: demographic history inference with folded SNP frequency spectra. *Genome Biol* **21**: 1–9.

Maisano Delser P, Corrigan S, Hale M, Li C, Veuille M, Planes S, *et al.* (2016). Population genomics of C. melanopterus using target gene capture data: Demographic inferences and conservation perspectives. *Sci Rep* **6**: 1–12.

Rochette NC, Rivera-Colón AG, Catchen JM (2019). Stacks 2: Analytical methods for paired-end sequencing improve RADseq-based population genomics. *Mol Ecol* **28**: 4737–4754.

Walsh CAJ, Momigliano P, Boussarie G, Robbins WD, Bonnin L, Fauvelot C, *et al.* (2022). Genomic insights into the historical and contemporary demographics of the grey reef shark.

Supplementary Tables

Table S1. Confusion matrix of the model selection procedure: rows indicate the simulated models and columns the votes (in %) attributed by the ABC-RF algorithm to each of them.

		Attributed votes (%)			Class. error
		FIM	NS	SST	
Juan	FIM	41396	2027	6577	0.17
	NS	663	48706	631	0.03
	SST	5813	1059	43128	0.14
Bampton	FIM	40105	2251	7644	0.2
	NS	719	48590	691	0.03
	SST	6949	1432	41619	0.17
Belep	FIM	38085	2537	9378	0.24
	NS	822	48275	903	0.03
	SST	8790	1745	39465	0.21
Enderbury	FIM	41148	2141	6711	0.18
	NS	662	48637	701	0.03
	SST	5939	1091	42970	0.14
Kanton	FIM	40262	2269	7469	0.19
	NS	758	48507	735	0.03
	SST	6821	1366	41813	0.16
McKean	FIM	37984	2613	9403	0.24
	NS	837	48299	864	0.03
	SST	8825	1752	39423	0.21
Niku	FIM	42260	1851	5889	0.15
	NS	566	48925	509	0.02
	SST	4988	795	44217	0.12
Orona	FIM	40395	2203	7402	0.19
	NS	705	48595	700	0.03
	SST	6538	1284	42178	0.16
Palmyra	FIM	43083	1611	5306	0.14
	NS	483	49113	404	0.02
	SST	4344	561	45095	0.1
Fakarava	FIM	42156	1887	5957	0.16
	NS	607	48829	564	0.02
	SST	5140	870	43990	0.12

Table S2. Cross-validation of the ABC-RF procedure of the SST model: Mean Squared Error (SME), Mean Root Squared Error (RMSE) and 95% coverage of the median value for each parameter computed on 999 pseudo-observed datasets (pods).

		Nm	T_{col}	N_{anc}
Juan	Coverage	0.997	1	0.996
	SME	0.00448424	0.01473625	0.01679217
	MRSE	0.06559084	0.15917352	0.20927024
Bampton	Coverage	0.996	0.993	0.996
	SME	0.00418188	0.14437886	0.03688622
	MRSE	0.03547396	2.89427943	0.37985902
Belep	Coverage	0.997	0.994	0.998
	SME	0.00607304	0.06137466	0.0207203
	MRSE	0.08473424	0.78386441	0.17434075
Enderbury	Coverage	0.996	0.996	0.997
	SME	0.00179036	0.13584242	0.04827846
	MRSE	0.02448663	2.86874802	0.73693169
Kanton	Coverage	0.99	0.993	0.997
	SME	0.00153847	0.06132533	0.08600365
	MRSE	0.03178348	0.63525529	1.30039719
Mckean	Coverage	0.992	0.994	0.99
	SME	0.00563147	0.11468267	0.06404819
	MRSE	0.06642286	1.13569438	0.92112944
Niku	Coverage	0.999	0.999	0.999
	SME	0.00220155	0.03579402	0.01821552
	MRSE	0.03032898	0.47453067	0.25503461
Orona	Coverage	0.998	0.997	0.996
	SME	0.00489788	0.02427145	0.04237548
	MRSE	0.04727987	0.27677012	0.38470955
Palmyra	Coverage	0.999	0.998	0.998
	SME	0.00012809	0.01689414	0.02611148
	MRSE	0.01099299	0.27849299	0.45547058
Fakarava	Coverage	0.999	0.997	0.997
	SME	0.00304558	0.10564623	0.0475489
	MRSE	0.02769755	2.11211995	0.55497854

Table S3. Matrix of pairwise F_{ST} values (lower triangle) and associated p-value (upper triangle). Color represents the region of origin: Indian ocean (yellow), Chesterfield islands (red), New Caledonia (green), Phoenix islands (blue), Palmyra (cyan) and French polynesia (pink).

	Juan	Zelee	Bampton	Avond	Belep	Poindimie	Niku	Mckean	Orona	Kanton	Enderbury	Palmyra	Moorea	Fakarava
Juan		NS	$p \leq 0.001$	$p \leq 0.001$	$p \leq 0.001$	$p \leq 0.001$	$p \leq 0.001$	$p \leq 0.001$	$p \leq 0.001$	$p \leq 0.001$	$p \leq 0.001$	$p \leq 0.001$	$p \leq 0.001$	$p \leq 0.001$
Zelee	0.0003		$p \leq 0.001$	$p \leq 0.001$	$p \leq 0.001$	$p \leq 0.001$	$p \leq 0.001$	$p \leq 0.001$	$p \leq 0.001$	$p \leq 0.001$	$p \leq 0.001$	$p \leq 0.001$	$p \leq 0.001$	$p \leq 0.001$
Bampton	0.5351	0.5373		NS	$p \leq 0.001$	$p \leq 0.001$	$p \leq 0.001$	$p \leq 0.001$	$p \leq 0.001$	$p \leq 0.001$	$p \leq 0.001$	$p \leq 0.001$	$p \leq 0.001$	$p \leq 0.001$
Avond	0.5298	0.5324	0.0001		$p \leq 0.001$	NS	$p \leq 0.001$	$p \leq 0.001$	$p \leq 0.001$	$p \leq 0.001$	$p \leq 0.001$	$p \leq 0.001$	$p \leq 0.001$	$p \leq 0.001$
Belep	0.5271	0.5296	0.0069	0.0046		NS	$p \leq 0.001$	$p \leq 0.001$	$p \leq 0.001$	$p \leq 0.001$	$p \leq 0.001$	$p \leq 0.001$	$p \leq 0.001$	$p \leq 0.001$
Poindimie	0.5302	0.5327	0.0078	0.0073	0.0013		$p \leq 0.001$	$p \leq 0.001$	$p \leq 0.001$	$p \leq 0.001$	$p \leq 0.001$	$p \leq 0.001$	$p \leq 0.001$	$p \leq 0.001$
Niku	0.5378	0.5402	0.0178	0.0163	0.0111	0.014		NS	NS	NS	NS	$p \leq 0.001$	$p \leq 0.001$	$p \leq 0.001$
Mckean	0.5379	0.5404	0.0173	0.0169	0.0119	0.013	0.0004		NS	NS	NS	NS	$p \leq 0.001$	$p \leq 0.001$
Orona	0.5434	0.5459	0.0172	0.0177	0.0117	0.0135	0.0009	0.0005		NS	NS	$p \leq 0.001$	$p \leq 0.001$	$p \leq 0.001$
Kanton	0.5493	0.5519	0.0162	0.016	0.0117	0.0126	0.0003	0.0012	0.001		NS	NS	$p \leq 0.001$	$p \leq 0.001$
Enderbury	0.5352	0.538	0.0167	0.0163	0.0118	0.0131	0.0008	0.0007	0.0019	0.0003		$p \leq 0.001$	NS	$p \leq 0.001$
Palmyra	0.5419	0.5444	0.0207	0.0194	0.0147	0.0159	0.0034	0.0023	0.0045	0.0016	0.0036		$p \leq 0.001$	$p \leq 0.001$
Moorea	0.5473	0.5499	0.0263	0.028	0.0218	0.0228	0.0109	0.0099	0.0102	0.0078	0.0125	0.0119		NS
Fakarava	0.5549	0.5575	0.024	0.0238	0.0215	0.0228	0.0097	0.0089	0.0084	0.0061	0.0101	0.01	0.0049	

Supplementary Figures

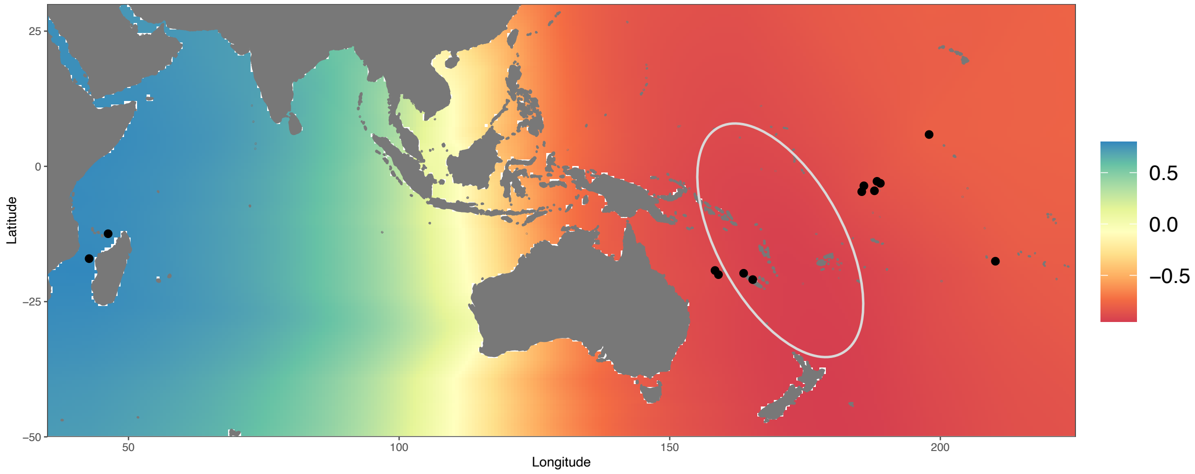

Figure S1. Correlation map between genetic diversity (θ_π) and Least Cost (LC) distances when considering all sampling sites. Each cell is coloured according to the correlation coefficient value computed between θ_π and the LC distance from the putative origin of the range expansion (RE). Black dots represent the sampling sites considered.

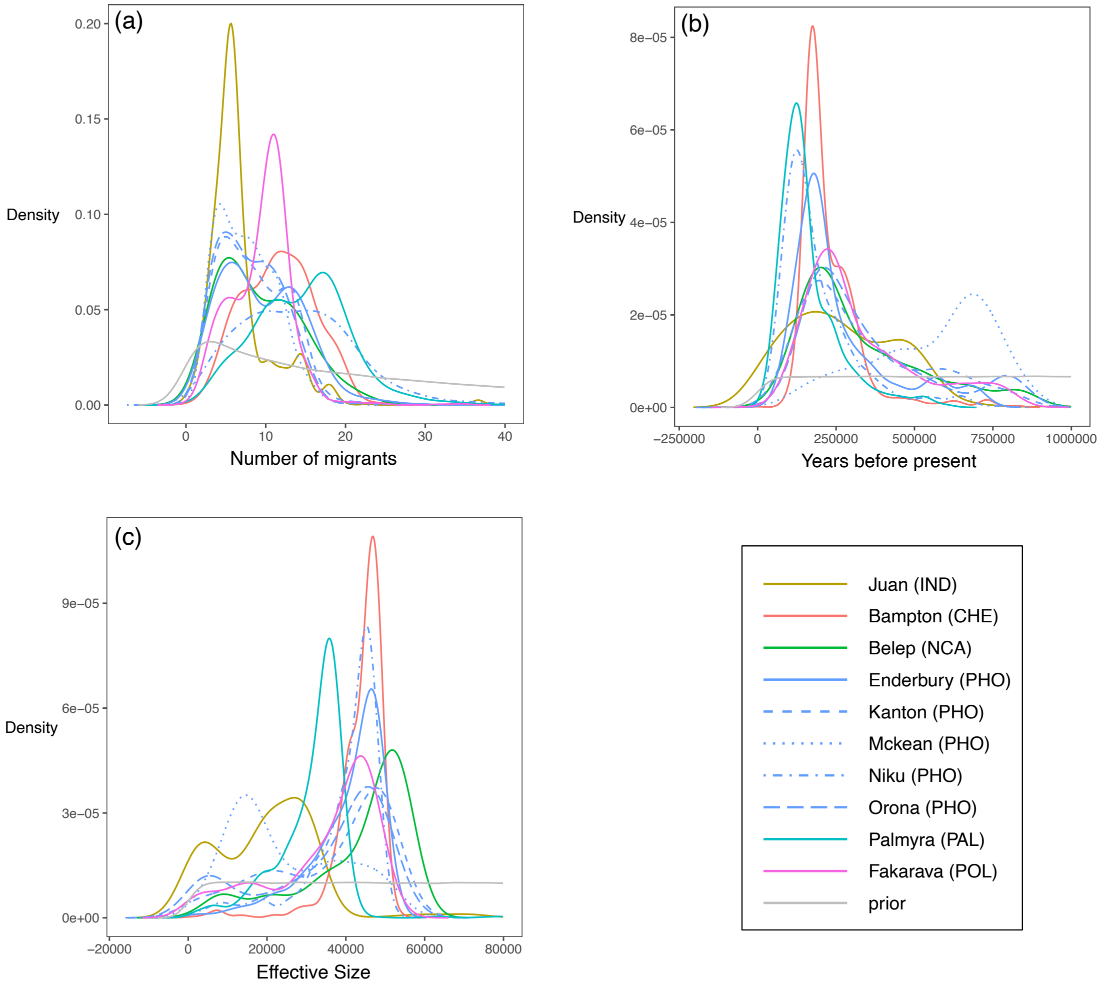

Figure S2. Posterior distribution of the number of migrants per generation Nm (a), the colonisation time of the array of deme T_{col} (b) and of the ancestral effective size N_{anc} (c) estimated under the stepping stone model (SST) for all sampling sites with $N_{ind} \geq 7$. Colours represent the origin of the populations: Indian Ocean (yellow), Chesterfield islands (red), New Caledonia (green), Phoenix islands (blue), Palmyra (cyan) and Polynesia (purple). Line types represent the different populations from the Phoenix islands: Enderbury (solid), Kanton (dashes), Mckean (dots), Niku (dot-dashes) and Orona (long-dashes). The prior distribution is coloured in grey.

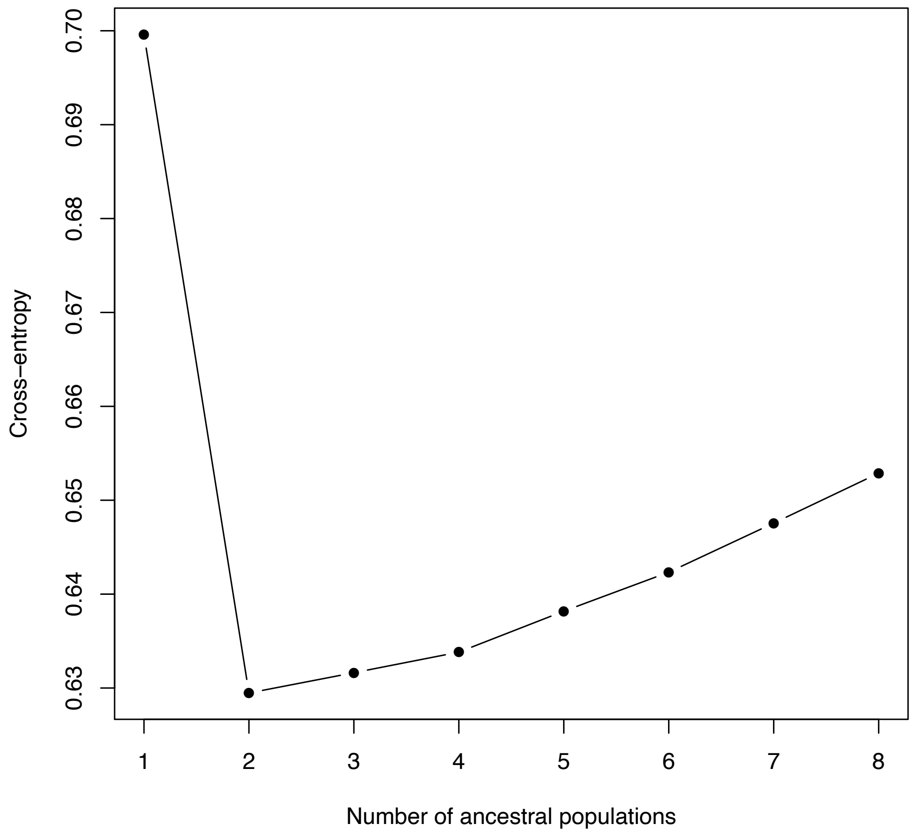

Figure S3. Cross entropy criterion of the *sNMF* algorithm computed for K=1 to K=8 ancestral populations.

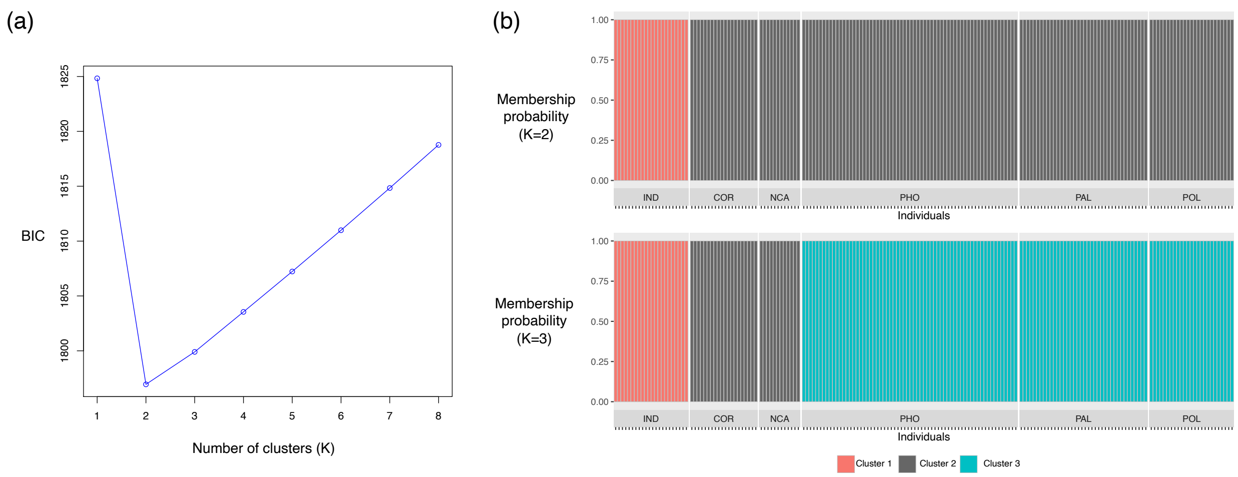

Figure S4. Results of the Discriminant Analysis of Principal Components. Bayesian Information Criterion (BIC) computed from K=1 to K=8 clusters (a) and membership probability of each individual to the clusters when considering K = 2 or K = 3 (b).

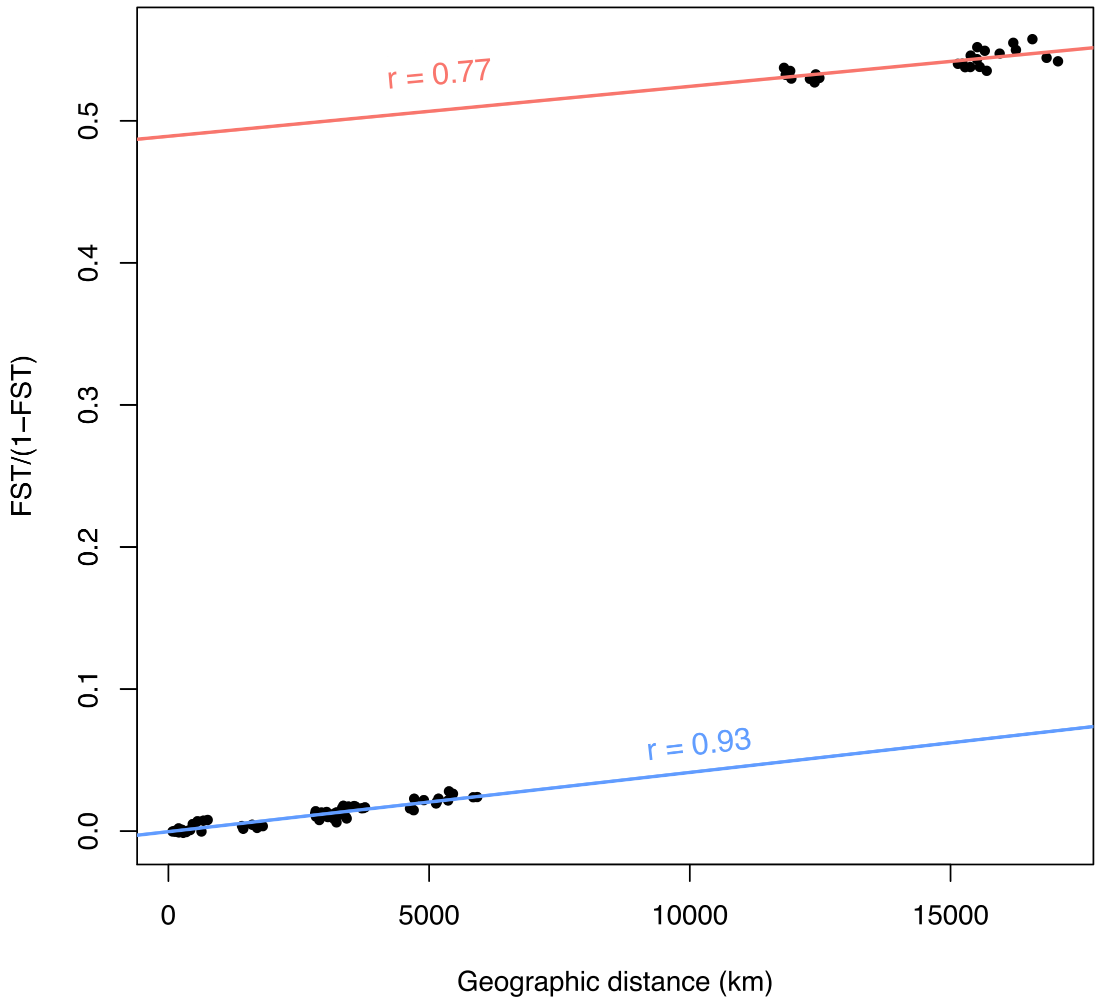

Figure S5. Isolation by distance (IBD) plot with all sampling sites. Correlation value and regression line computed between genetic and geographic distances when considering only Indian *vs.* Pacific sampling sites (red) or when considering only Pacific sampling sites (blue).

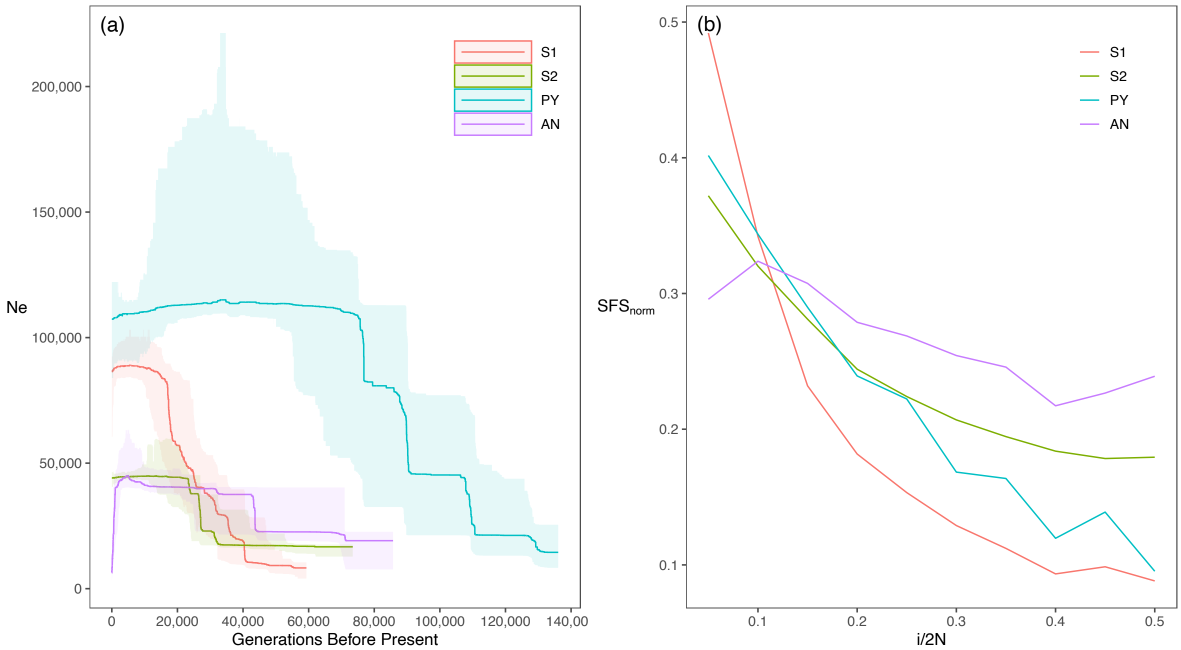

Figure S6. Variation of the effective population size (*Ne*) through time and its 75% confidence interval estimated by the *stairwayplot* (a) and Normalized Site Frequency Spectrum (b) of Bampton site (N=10) computed from data assembled using the different variant calling pipelines: ANGSD (AN, purple), STACKS v.2.5 (S2, green), STACKS v.1.48 (S1, red) and Pyrad (PY, blue). The *stairwayplot* was computed using the mutation rate μ=1.9434e-08 per site per generation and a generation time of 16.4 years as in Walsh *et al.*, (2022).

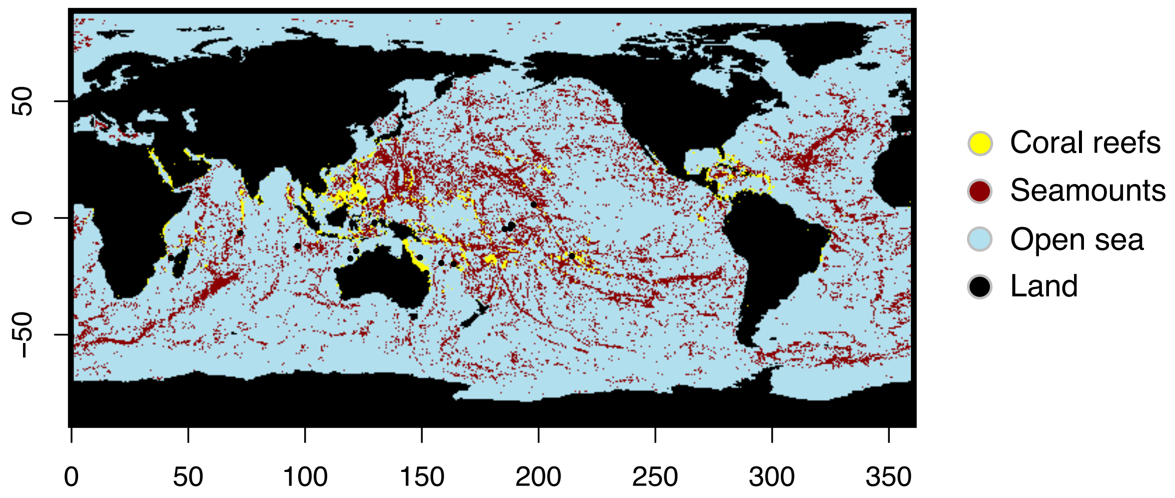

Figure S7. Distribution of corals and seamounts in the Indo-Pacific oceans. Cells are coloured according to their habitat type: coral reefs (yellow), seamounts (red), open sea (blue) and land (black).

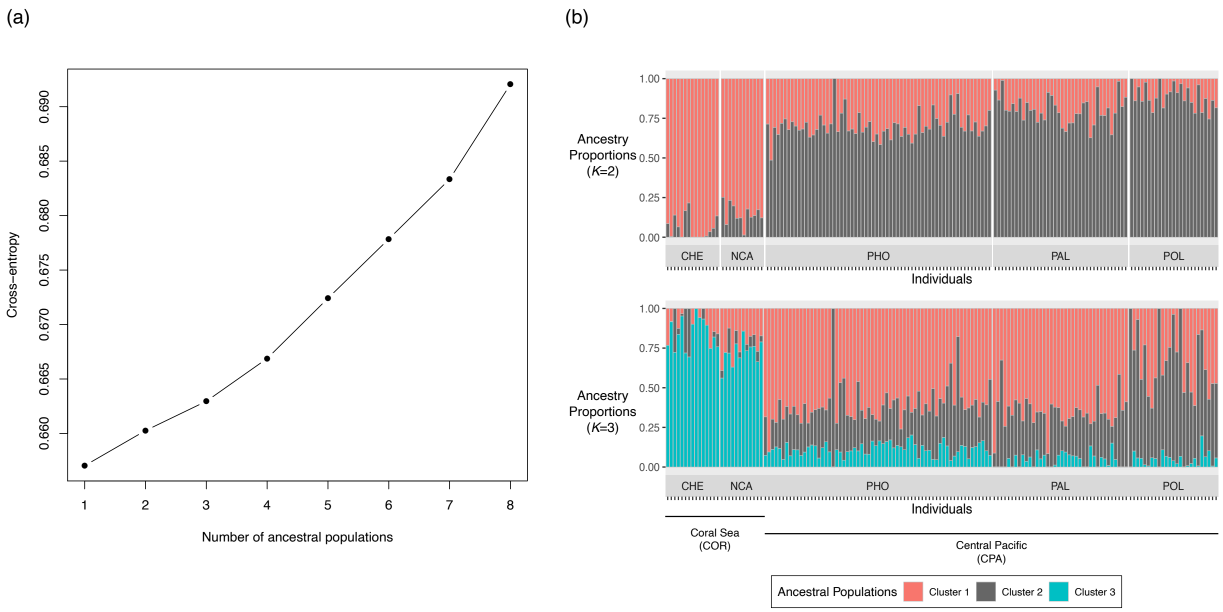

Figure S8. *sNMF* algorithm computed on Pacific samples only. Cross entropy criterion of the *sNMF* algorithm computed for K=1 to K=8 ancestral populations (a) and ancestry proportions retrieved with *K*=2 *and* K=*3* ancestral populations (b).
